# 7-Bromo-2-methyl-1-tosyl­naphtho[2,1-*b*]furan

**DOI:** 10.1107/S1600536808015286

**Published:** 2008-05-24

**Authors:** Hong Dae Choi, Pil Ja Seo, Byeng Wha Son, Uk Lee

**Affiliations:** a Department of Chemistry, Dongeui University, San 24 Kaya-dong Busanjin-gu, Busan 614-714, Republic of Korea; b Department of Chemistry, Pukyong National University, 599-1 Daeyeon 3-dong Nam-gu, Busan 608-737, Republic of Korea

## Abstract

The title compound, C_20_H_15_BrO_3_S, was prepared by the oxidation of 7-bromo-2-methyl-1-(4-tolyl­sulfan­yl)naph­tho[2,1-*b*]furan with 3-chloro­peroxy­benzoic acid. The 4-tolyl ring makes a dihedral angle of 70.96 (6)° with the plane of the naphthofuran fragment. The crystal structure is stabilized by aromatic π–π stacking inter­actions, with centroid–centroid distances of 3.672 (3) and 3.858 (3) Å between the central benzene and furan rings, and between the brominated benzene and central benzene rings of the naphthofuran system of neighbouring mol­ecules, respectively. In addition, the stacked mol­ecules exhibit C—H⋯π and inter- and intra­molecular C—H⋯O inter­actions.

## Related literature

For the crystal structures of similar 2-methyl-1-(phenyl­sulfon­yl)naphtho[2,1-*b*]furan compounds, see: Choi *et al.* (2008*a* ▶,*b* ▶).
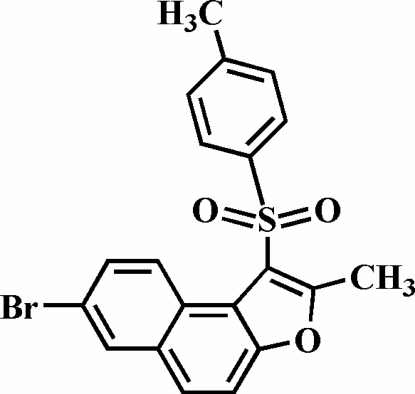


## Experimental

### 

#### Crystal data

C_20_H_15_BrO_3_S
*M* *_r_* = 415.29
Monoclinic, 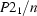

*a* = 14.026 (2) Å
*b* = 8.225 (1) Å
*c* = 15.185 (2) Å
β = 102.826 (2)°
*V* = 1708.1 (4) Å^3^
*Z* = 4
Mo *K*α radiation
μ = 2.54 mm^−1^
*T* = 173 (2) K
0.30 × 0.30 × 0.20 mm

#### Data collection

Bruker SMART CCD diffractometer
Absorption correction: multi-scan (*SADABS*; Sheldrick, 2000 ▶) *T*_min_ = 0.480, *T*_max_ = 0.608
10014 measured reflections
3704 independent reflections
3368 reflections with *I* > 2σ(*I*)
*R*_int_ = 0.024

#### Refinement

*R*[*F* ^2^ > 2σ(*F* ^2^)] = 0.032
*wR*(*F* ^2^) = 0.082
*S* = 1.09
3704 reflections
228 parameters
H-atom parameters constrained
Δρ_max_ = 0.61 e Å^−3^
Δρ_min_ = −1.04 e Å^−3^

### 

Data collection: *SMART* (Bruker, 2001 ▶); cell refinement: *SAINT* (Bruker, 2001 ▶); data reduction: *SAINT*; program(s) used to solve structure: *SHELXS97* (Sheldrick, 2008 ▶); program(s) used to refine structure: *SHELXL97* (Sheldrick, 2008 ▶); molecular graphics: *ORTEP-3* (Farrugia, 1997 ▶) and *DIAMOND* (Brandenburg, 1998 ▶); software used to prepare material for publication: *SHELXL97*.

## Supplementary Material

Crystal structure: contains datablocks global, I. DOI: 10.1107/S1600536808015286/at2569sup1.cif

Structure factors: contains datablocks I. DOI: 10.1107/S1600536808015286/at2569Isup2.hkl

Additional supplementary materials:  crystallographic information; 3D view; checkCIF report

## Figures and Tables

**Table 1:** Hydrogen-bond geometry (Å, °)

*D*—H⋯*A*	*D*—H	H⋯*A*	*D*⋯*A*	*D*—H⋯*A*
C10—H10⋯*Cg*1^i^	0.95	2.57	3.485 (3)	163
C4—H4⋯O2	0.95	2.21	3.035 (2)	145
C15—H15⋯O3^ii^	0.95	2.42	3.303 (2)	155

Symmetry codes: (i) 
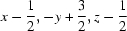
; (ii) 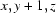. *Cg*1 is the centroid of the C13–C18 benzene ring.

**Fig. 1.**
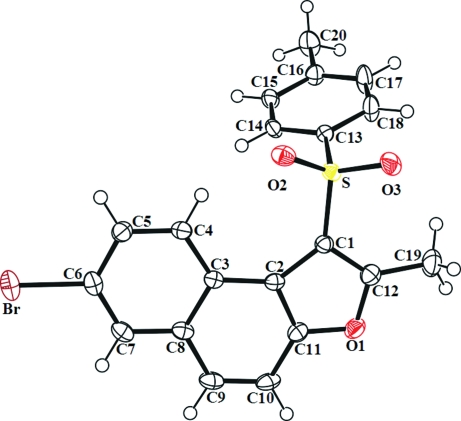
The molecular structure of the title compound, showing displacement ellipsoids drawn at the 50% probability level.

**Fig. 2.**
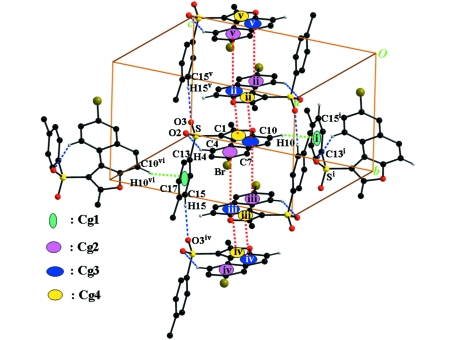
π···π, C—H···π and C—H···O interactions (dotted lines) in the title compound. Cg denotes the ring centroids. [Symmetry codes: (i) x-1/2, -y+3/2, z-1/2; (ii) -x+1, -y+1, -z+1; (iii) -x+1, -y+2, -z+1; (iv) x, y+1, z; (v) x, y-1, z; (vi) x+1/2, -y+3/2, z+1/2.]

## supplementary crystallographic information

### Comment

This work is related to our communications on the synthesis and structures of 2-methyl-1-(phenylsulfonyl)naphtho[2,1-b]furan analogues, viz. 2-methyl-1-(phenylsulfonyl)naphtho[2,1-b]furan (Choi *et al.*, 2008a) and 7-bromo-2-methyl-1-(phenylsulfonyl)naphtho[2,1-b]furan (Choi *et al.*, 2008b). Here we report the crystal structure of the title compound, 7-bromo-2-methyl-1-tosylnaphtho[2,1-b]furan (Fig. 1).

The naphthofuran unit is essentially planar, with a mean deviation of 0.01 Å from the least-squares plane defined by the thirteen constituent atoms. The 4-tolyl ring (C13-C18) makes a dihedral angle of 70.96 (6)° with the plane of the naphthofuran fragment. The molecular packing (Fig. 2) is stabilized by two different π—π interactions within each stack of molecule; one between the central benzene ring (Cg3) and the furan ring (Cg4^ii^) of the adjacent naphthofuran fragments {distance; 3.672 (3) Å}, and a second between the brominated benzene ring (Cg2) and the central benzene ring (Cg3^iii^) of the adjacent naphthofuran fragments {distance; 3.858 (3) Å} (Fig. 2; Cg2, Cg3 and Cg4 are the centroids of the C3-C8 benzene, the C2/C3/C8/C9/C10/C11 benzene, and the O1/C12/C1/C2/C11 furan rings, respectively, symmetry code as in Fig. 2). The crystal packing is further stabilized by C—H···π interaction between a central benzene H atom of naphthofuran unit and the 4-tolyl ring of the tosyl substituent, with a C10—H10···Cg1^i^ separation of 2.57 Å (Fig. 2 and Table 1; Cg1 is the centroid of the C13-C18 phenyl ring; symmetry code as in Fig. 2). Additionally, inter- and intramolecular C—H···O interactions in the structure were observed (Fig. 2 and Table 1; symmetry code as in Fig. 2).

### Experimental

3-Chloroperoxybenzoic acid (77%, 377 mg, 1.68 mmol) was added in small portions to a stirred solution of 7-bromo-2-methyl-1-(4-tolylsulfanyl)naphtho[2,1-b]furan (306 mg, 0.8 mmol) in dichloromethane (30 ml) at 273 K. After being stirred at room temperature for 4 h, the mixture was washed with saturated sodium bicarbonate solution and the organic layer was separated, dried over magnesium sulfate, filtered and concentrated in vacuum. The residue was purified by column chromatography (chloroform) to afford the title compound as a colourless solid [yield 79%, m.p. 480-481 K; R_f_ = 0.51 (chloroform)]. Single crystals suitable for X-ray diffraction were prepared by evaporation of a solution of the title compound in chloroform at room temperature. Spectroscopic analysis: ^1^H NMR (CDCl_3_, 400 MHz) δ 2.35 (s, 3H), 2.99 (s, 3H), 7.27 (s, 2H), 7.59-7.68 (m, 3H), 7.83 (d, J = 8.08 Hz, 2H), 8.03 (s, 1H), 8.91 (s, J = 9.16 Hz, 1H); EI-MS 416 [M+2], 414 [M^+^].

### Refinement

All H atoms were geometrically positioned and refined using a riding model, with C—H = 0.95 Å for aromatic H atoms, 0.98 Å for methyl H atoms, respectively, and with U_iso_(H) = 1.2U_eq_(C) for aromatic H atoms and 1.5U_eq_(C) for methyl H atoms. The highest peak in the difference map is 0.77 Å from Br and the largest hole is 0.67 Å from Br.

### Crystal data

**Table:**

C_20_H_15_BrO_3_S	*F*_000_ = 840
*M**_r_* = 415.29	*D*_x_ = 1.615 Mg m^−^^3^
Monoclinic, *P*2_1_/*n*	Melting point = 480–481 K
Hall symbol: -P 2yn	Mo *K*α radiation λ = 0.71073 Å
*a* = 14.026 (2) Å	Cell parameters from 6484 reflections
*b* = 8.225 (1) Å	θ = 2.2–28.3º
*c* = 15.185 (2) Å	µ = 2.55 mm^−^^1^
β = 102.826 (2)º	*T* = 173 (2) K
*V* = 1708.1 (4) Å^3^	Block, colourless
*Z* = 4	0.30 × 0.30 × 0.20 mm

### Data collection

**Table:**

Bruker SMART CCD diffractometer	3704 independent reflections
Radiation source: fine-focus sealed tube	3368 reflections with *I* > 2σ(*I*)
Monochromator: graphite	*R*_int_ = 0.024
Detector resolution: 10 pixels mm^-1^	θ_max_ = 27.0º
*T* = 173(2) K	θ_min_ = 2.8º
φ and ω scans	*h* = −17→14
Absorption correction: multi-scan(SADABS; Sheldrick, 2000)	*k* = −10→9
*T*_min_ = 0.480, *T*_max_ = 0.608	*l* = −19→19
10014 measured reflections	

### Refinement

**Table:**

Refinement on *F*^2^	Secondary atom site location: difference Fourier map
Least-squares matrix: full	Hydrogen site location: inferred from neighbouring sites
*R*[*F*^2^ > 2σ(*F*^2^)] = 0.032	H-atom parameters constrained
*wR*(*F*^2^) = 0.082	*w* = 1/[σ^2^(*F*_o_^2^) + (0.0373*P*)^2^ + 1.421*P*] where *P* = (*F*_o_^2^ + 2*F*_c_^2^)/3
*S* = 1.09	(Δ/σ)_max_ = 0.001
3704 reflections	Δρ_max_ = 0.61 e Å^−^^3^
228 parameters	Δρ_min_ = −1.04 e Å^−^^3^
Primary atom site location: structure-invariant direct methods	Extinction correction: none

### Special details

**Table:**

Geometry. All esds (except the esd in the dihedral angle between two l.s. planes) are estimated using the full covariance matrix. The cell esds are taken into account individually in the estimation of esds in distances, angles and torsion angles; correlations between esds in cell parameters are only used when they are defined by crystal symmetry. An approximate (isotropic) treatment of cell esds is used for estimating esds involving l.s. planes.
Refinement. Refinement of F^2^ against ALL reflections. The weighted R-factor wR and goodness of fit S are based on F^2^, conventional R-factors R are based on F, with F set to zero for negative F^2^. The threshold expression of F^2^ > 2sigma(F^2^) is used only for calculating R-factors(gt) etc. and is not relevant to the choice of reflections for refinement. R-factors based on F^2^ are statistically about twice as large as those based on F, and R- factors based on ALL data will be even larger.

### Fractional atomic coordinates and isotropic or equivalent isotropic displacement parameters (Å^2^)

**Table:**

	*x*	*y*	*z*	*U*_iso_*/*U*_eq_	
Br	0.307602 (17)	1.04128 (3)	0.664085 (18)	0.04012 (10)	
S	0.79201 (3)	0.59282 (6)	0.62949 (3)	0.01860 (11)	
O1	0.64155 (11)	0.52776 (18)	0.38321 (9)	0.0264 (3)	
O2	0.74469 (10)	0.61119 (18)	0.70363 (9)	0.0248 (3)	
O3	0.85357 (11)	0.45249 (17)	0.62854 (11)	0.0276 (3)	
C1	0.70375 (14)	0.5926 (2)	0.52785 (12)	0.0190 (4)	
C2	0.60466 (14)	0.6593 (2)	0.50343 (12)	0.0184 (4)	
C3	0.53912 (13)	0.7504 (2)	0.54485 (12)	0.0177 (4)	
C4	0.56078 (14)	0.8095 (2)	0.63474 (13)	0.0214 (4)	
H4	0.6233	0.7883	0.6723	0.026*	
C5	0.49378 (15)	0.8966 (3)	0.66890 (14)	0.0245 (4)	
H5	0.5102	0.9368	0.7290	0.029*	
C6	0.40102 (15)	0.9256 (3)	0.61444 (15)	0.0249 (4)	
C7	0.37592 (15)	0.8727 (3)	0.52738 (14)	0.0250 (4)	
H7	0.3125	0.8947	0.4917	0.030*	
C8	0.44413 (14)	0.7849 (2)	0.48995 (13)	0.0209 (4)	
C9	0.41645 (15)	0.7306 (3)	0.39838 (14)	0.0266 (4)	
H9	0.3529	0.7550	0.3638	0.032*	
C10	0.47904 (16)	0.6451 (3)	0.35995 (13)	0.0264 (4)	
H10	0.4611	0.6088	0.2991	0.032*	
C11	0.57167 (15)	0.6128 (2)	0.41433 (13)	0.0219 (4)	
C12	0.72149 (16)	0.5171 (2)	0.45281 (14)	0.0241 (4)	
C13	0.86530 (13)	0.7660 (2)	0.62557 (12)	0.0181 (4)	
C14	0.82844 (14)	0.9193 (2)	0.63701 (13)	0.0207 (4)	
H14	0.7635	0.9315	0.6449	0.025*	
C15	0.88778 (15)	1.0550 (2)	0.63683 (14)	0.0229 (4)	
H15	0.8626	1.1602	0.6441	0.027*	
C16	0.98325 (16)	1.0390 (2)	0.62625 (15)	0.0249 (4)	
C17	1.01851 (17)	0.8839 (3)	0.61549 (19)	0.0361 (5)	
H17	1.0837	0.8713	0.6083	0.043*	
C18	0.96049 (16)	0.7473 (3)	0.61504 (17)	0.0309 (5)	
H18	0.9856	0.6421	0.6076	0.037*	
C19	0.80537 (19)	0.4294 (3)	0.43022 (17)	0.0359 (5)	
H19A	0.8032	0.3148	0.4473	0.054*	
H19B	0.8667	0.4782	0.4633	0.054*	
H19C	0.8017	0.4374	0.3652	0.054*	
C20	1.04846 (17)	1.1859 (3)	0.62979 (18)	0.0360 (5)	
H20A	1.0081	1.2836	0.6153	0.054*	
H20B	1.0896	1.1731	0.5858	0.054*	
H20C	1.0901	1.1963	0.6906	0.054*	

### Atomic displacement parameters (Å^2^)

**Table:**

	*U*^11^	*U*^22^	*U*^33^	*U*^12^	*U*^13^	*U*^23^
Br	0.03043 (14)	0.04477 (17)	0.04947 (17)	0.01043 (10)	0.01810 (11)	−0.00261 (11)
S	0.0176 (2)	0.0165 (2)	0.0206 (2)	0.00131 (16)	0.00179 (17)	0.00252 (17)
O1	0.0320 (8)	0.0272 (8)	0.0197 (7)	0.0013 (6)	0.0050 (6)	−0.0045 (6)
O2	0.0238 (7)	0.0306 (8)	0.0191 (7)	−0.0015 (6)	0.0029 (5)	0.0053 (6)
O3	0.0259 (8)	0.0169 (7)	0.0370 (8)	0.0048 (6)	0.0009 (6)	0.0022 (6)
C1	0.0194 (9)	0.0179 (9)	0.0191 (9)	0.0000 (7)	0.0030 (7)	−0.0002 (7)
C2	0.0204 (9)	0.0149 (8)	0.0183 (9)	−0.0019 (7)	0.0008 (7)	0.0014 (7)
C3	0.0180 (9)	0.0155 (8)	0.0187 (8)	−0.0011 (7)	0.0021 (7)	0.0026 (7)
C4	0.0187 (9)	0.0241 (10)	0.0201 (9)	−0.0014 (7)	0.0015 (7)	−0.0001 (7)
C5	0.0242 (10)	0.0264 (10)	0.0233 (10)	−0.0020 (8)	0.0061 (8)	−0.0034 (8)
C6	0.0219 (10)	0.0225 (10)	0.0324 (11)	0.0021 (8)	0.0107 (8)	0.0020 (8)
C7	0.0189 (9)	0.0252 (10)	0.0288 (10)	0.0024 (8)	0.0009 (8)	0.0058 (8)
C8	0.0206 (9)	0.0189 (9)	0.0211 (9)	−0.0016 (7)	0.0003 (7)	0.0040 (7)
C9	0.0244 (10)	0.0276 (10)	0.0227 (10)	−0.0006 (8)	−0.0056 (8)	0.0040 (8)
C10	0.0323 (11)	0.0268 (10)	0.0159 (9)	−0.0024 (8)	−0.0041 (8)	−0.0007 (8)
C11	0.0259 (10)	0.0193 (9)	0.0205 (9)	−0.0012 (8)	0.0048 (8)	−0.0005 (7)
C12	0.0263 (10)	0.0219 (10)	0.0243 (10)	−0.0001 (8)	0.0059 (8)	0.0005 (8)
C13	0.0176 (9)	0.0179 (9)	0.0184 (8)	0.0008 (7)	0.0029 (7)	−0.0005 (7)
C14	0.0157 (9)	0.0213 (9)	0.0245 (9)	0.0040 (7)	0.0029 (7)	0.0011 (7)
C15	0.0224 (10)	0.0177 (9)	0.0274 (10)	0.0041 (7)	0.0030 (8)	0.0005 (7)
C16	0.0248 (10)	0.0216 (10)	0.0295 (10)	−0.0020 (8)	0.0085 (8)	−0.0016 (8)
C17	0.0246 (11)	0.0275 (12)	0.0625 (16)	0.0002 (9)	0.0231 (11)	−0.0077 (11)
C18	0.0260 (11)	0.0201 (10)	0.0507 (13)	0.0019 (8)	0.0170 (10)	−0.0063 (9)
C19	0.0361 (13)	0.0377 (13)	0.0379 (12)	0.0065 (10)	0.0166 (10)	−0.0062 (10)
C20	0.0276 (11)	0.0271 (11)	0.0563 (15)	−0.0058 (9)	0.0158 (11)	−0.0028 (10)

### Geometric parameters (Å, °)

**Table:**

Br—C6	1.905 (2)	C9—H9	0.9500
S—O2	1.436 (2)	C10—C11	1.401 (3)
S—O3	1.443 (2)	C10—H10	0.9500
S—C1	1.751 (2)	C12—C19	1.483 (3)
S—C13	1.765 (2)	C13—C18	1.388 (3)
O1—C12	1.362 (3)	C13—C14	1.388 (3)
O1—C11	1.371 (3)	C14—C15	1.392 (3)
C1—C12	1.368 (3)	C14—H14	0.9500
C1—C2	1.463 (3)	C15—C16	1.390 (3)
C2—C11	1.383 (3)	C15—H15	0.9500
C2—C3	1.435 (3)	C16—C17	1.391 (3)
C3—C4	1.417 (3)	C16—C20	1.509 (3)
C3—C8	1.434 (3)	C17—C18	1.387 (3)
C4—C5	1.372 (3)	C17—H17	0.9500
C4—H4	0.9500	C18—H18	0.9500
C5—C6	1.399 (3)	C19—H19A	0.9800
C5—H5	0.9500	C19—H19B	0.9800
C6—C7	1.362 (3)	C19—H19C	0.9800
C7—C8	1.415 (3)	C20—H20A	0.9800
C7—H7	0.9500	C20—H20B	0.9800
C8—C9	1.430 (3)	C20—H20C	0.9800
C9—C10	1.354 (3)		
			
O2—S—O3	118.26 (9)	O1—C11—C2	111.6 (2)
O2—S—C1	109.37 (9)	O1—C11—C10	122.3 (2)
O3—S—C1	107.33 (9)	C2—C11—C10	126.1 (2)
O2—S—C13	108.39 (9)	O1—C12—C1	110.3 (2)
O3—S—C13	106.92 (9)	O1—C12—C19	114.2 (2)
C1—S—C13	105.90 (9)	C1—C12—C19	135.5 (2)
C12—O1—C11	107.1 (2)	C18—C13—C14	120.7 (2)
C12—C1—C2	107.3 (2)	C18—C13—S	119.8 (2)
C12—C1—S	120.7 (2)	C14—C13—S	119.5 (1)
C2—C1—S	132.0 (2)	C13—C14—C15	119.2 (2)
C11—C2—C3	117.7 (2)	C13—C14—H14	120.4
C11—C2—C1	103.7 (2)	C15—C14—H14	120.4
C3—C2—C1	138.6 (2)	C16—C15—C14	121.1 (2)
C4—C3—C8	117.7 (2)	C16—C15—H15	119.5
C4—C3—C2	125.5 (2)	C14—C15—H15	119.5
C8—C3—C2	116.8 (2)	C15—C16—C17	118.5 (2)
C5—C4—C3	121.7 (2)	C15—C16—C20	120.8 (2)
C5—C4—H4	119.2	C17—C16—C20	120.7 (2)
C3—C4—H4	119.2	C18—C17—C16	121.4 (2)
C4—C5—C6	119.4 (2)	C18—C17—H17	119.3
C4—C5—H5	120.3	C16—C17—H17	119.3
C6—C5—H5	120.3	C17—C18—C13	119.2 (2)
C7—C6—C5	121.7 (2)	C17—C18—H18	120.4
C7—C6—Br	119.5 (2)	C13—C18—H18	120.4
C5—C6—Br	118.9 (2)	C12—C19—H19A	109.5
C6—C7—C8	120.1 (2)	C12—C19—H19B	109.5
C6—C7—H7	120.0	H19A—C19—H19B	109.5
C8—C7—H7	120.0	C12—C19—H19C	109.5
C7—C8—C9	119.2 (2)	H19A—C19—H19C	109.5
C7—C8—C3	119.4 (2)	H19B—C19—H19C	109.5
C9—C8—C3	121.4 (2)	C16—C20—H20A	109.5
C10—C9—C8	121.3 (2)	C16—C20—H20B	109.5
C10—C9—H9	119.4	H20A—C20—H20B	109.5
C8—C9—H9	119.4	C16—C20—H20C	109.5
C9—C10—C11	116.7 (2)	H20A—C20—H20C	109.5
C9—C10—H10	121.6	H20B—C20—H20C	109.5
C11—C10—H10	121.6		
			
O2—S—C1—C12	−154.52 (16)	C12—O1—C11—C2	0.4 (2)
O3—S—C1—C12	−25.06 (19)	C12—O1—C11—C10	−179.39 (19)
C13—S—C1—C12	88.88 (18)	C3—C2—C11—O1	179.65 (16)
O2—S—C1—C2	23.9 (2)	C1—C2—C11—O1	−0.9 (2)
O3—S—C1—C2	153.39 (18)	C3—C2—C11—C10	−0.6 (3)
C13—S—C1—C2	−92.7 (2)	C1—C2—C11—C10	178.83 (19)
C12—C1—C2—C11	1.1 (2)	C9—C10—C11—O1	−179.86 (19)
S—C1—C2—C11	−177.49 (16)	C9—C10—C11—C2	0.4 (3)
C12—C1—C2—C3	−179.6 (2)	C11—O1—C12—C1	0.4 (2)
S—C1—C2—C3	1.8 (4)	C11—O1—C12—C19	−179.30 (18)
C11—C2—C3—C4	−179.21 (18)	C2—C1—C12—O1	−1.0 (2)
C1—C2—C3—C4	1.6 (4)	S—C1—C12—O1	177.83 (14)
C11—C2—C3—C8	0.3 (3)	C2—C1—C12—C19	178.7 (2)
C1—C2—C3—C8	−178.8 (2)	S—C1—C12—C19	−2.5 (4)
C8—C3—C4—C5	0.1 (3)	O2—S—C13—C18	133.70 (17)
C2—C3—C4—C5	179.65 (19)	O3—S—C13—C18	5.2 (2)
C3—C4—C5—C6	1.2 (3)	C1—S—C13—C18	−109.04 (18)
C4—C5—C6—C7	−1.5 (3)	O2—S—C13—C14	−43.50 (17)
C4—C5—C6—Br	178.18 (16)	O3—S—C13—C14	−172.00 (15)
C5—C6—C7—C8	0.4 (3)	C1—S—C13—C14	73.77 (17)
Br—C6—C7—C8	−179.22 (15)	C18—C13—C14—C15	0.7 (3)
C6—C7—C8—C9	−179.87 (19)	S—C13—C14—C15	177.89 (15)
C6—C7—C8—C3	0.9 (3)	C13—C14—C15—C16	−0.6 (3)
C4—C3—C8—C7	−1.1 (3)	C14—C15—C16—C17	0.2 (3)
C2—C3—C8—C7	179.28 (17)	C14—C15—C16—C20	−177.6 (2)
C4—C3—C8—C9	179.64 (18)	C15—C16—C17—C18	0.1 (4)
C2—C3—C8—C9	0.1 (3)	C20—C16—C17—C18	177.9 (2)
C7—C8—C9—C10	−179.5 (2)	C16—C17—C18—C13	0.0 (4)
C3—C8—C9—C10	−0.2 (3)	C14—C13—C18—C17	−0.4 (3)
C8—C9—C10—C11	0.0 (3)	S—C13—C18—C17	−177.58 (19)

### Hydrogen-bond geometry (Å, °)

**Table:**

*D*—H···*A*	*D*—H	H···*A*	*D*···*A*	*D*—H···*A*
C10—H10···Cg1^i^	0.95	2.57	3.485 (3)	163
C4—H4···O2	0.95	2.21	3.035 (2)	145
C15—H15···O3^ii^	0.95	2.42	3.303 (2)	155

Symmetry codes: (i) *x*−1/2, −*y*+3/2, *z*−1/2; (ii) *x*, *y*+1, *z*.
